# Development and Characterization of Intravenous Nanoemulsions Loaded with *Magnolia officinalis* Neolignans

**DOI:** 10.3390/molecules31111939

**Published:** 2026-06-03

**Authors:** Katarzyna Dominiak, Aleksandra Gostyńska-Stawna, Karina Sommerfeld-Klatta, Magdalena Ratajczak, Violetta Krajka-Kuźniak, Maciej Stawny

**Affiliations:** 1 Department of Pharmaceutical Chemistry, Poznan University of Medical Sciences, 3 Rokietnicka, 60-802 Poznan, Poland; katarzyna.dominiak@student.ump.edu.pl (K.D.); agostynska@ump.edu.pl (A.G.-S.); 2 Doctoral School, Poznan University of Medical Sciences, 70 Bukowska, 60-812 Poznan, Poland; 3 Department of Toxicology, Poznan University of Medical Sciences, 3 Rokietnicka, 60-806 Poznan, Poland; ksommerfeld@ump.edu.pl; 4 Department of Genetics and Pharmaceutical Microbiology, Poznan University of Medical Sciences, 3 Rokietnicka, 60-806 Poznan, Poland; mratajczak@ump.edu.pl; 5 Department of Pharmaceutical Biochemistry, Poznan University of Medical Sciences, 3 Rokietnicka, 60-806 Poznan, Poland; vkrajka@ump.edu.pl

**Keywords:** neolignans, nanoemulsion, parenteral nutrition, high-pressure homogenization, THLE-2 cells, PFAT5

## Abstract

Honokiol (HON) and magnolol (MAG), hepatoprotective neolignans from *Magnolia* spp., are promising candidates for mitigating liver damage associated with long-term parenteral nutrition (PN). However, their clinical use is limited by poor aqueous solubility. This study aimed to develop a multifunctional nanoemulsion platform based on clinically used lipid nanoemulsions (Lipofundin^®^/Lipidem^®^), enriched with omega-3 fatty acids and loaded with HON, MAG, or a combination of both. Nanoemulsions were prepared using a two-step homogenization process. Pre-emulsions containing HON, MAG, or both neolignans were first obtained using high-shear homogenization, then mixed in a 1:1 volume ratio with Lipofundin or Lipidem and subsequently subjected to high-pressure homogenization. All nanoemulsions met pharmacopeial standards and literature-recommended quality criteria. The formulations showed mean droplet diameters below 500 nm, PFAT5 values below the 0.05% limit, and PDI values < 0.2. Strongly negative zeta potentials (≤−30 mV) confirmed stability. The formulations were non-hemolytic, fully compatible with PN admixtures for 24 h, and non-cytotoxic in THLE-2 cells. Furthermore, all developed nanoemulsions demonstrated lower in vitro hepatotoxicity than clinically used reference formulations, indicating their promising clinical potential. However, further in vivo studies are required to confirm their therapeutic benefits and hepatoprotective effect during parenteral nutrition (PN).

## 1. Introduction

Intestinal failure-associated liver disease (IFALD) is a serious metabolic complication arising during long-term parenteral nutrition (PN). IFALD encompasses cholestasis, steatohepatitis, biliary cirrhosis, and gallbladder dysfunction. It affects up to 60–85% of children and 15–40% of adults receiving PN [1]. Its pathogenesis is multifactorial, with major contributors including phytosterols present in intravenous lipid emulsions, an unfavorable excess of omega-6 polyunsaturated fatty acids, and a concomitant deficiency of omega-3 fatty acids [2,3].

Intravenous lipid nanoemulsions are essential components of PN and serve as a major source of energy and essential fatty acids. Traditional soybean oil-based formulations contain high phytosterol levels, a predominance of omega-6 fatty acids, and relatively low omega-3 content [1]. When administered intravenously, phytosterols accumulate in hepatocytes, impair bile acid transport, and antagonize the farnesoid X receptor (FXR), a key regulator of cholesterol and bile acid homeostasis [4,5]. This contributes to cholestasis and dysregulation of lipid metabolism. Excess omega-6 fatty acids promote inflammation through arachidonic-acid-derived mediators, exacerbating hepatocyte injury [6]. In contrast, omega-3 fatty acids exert anti-inflammatory, antifibrotic, and lipid-modulating effects and have been shown to improve biochemical parameters and liver histology [7,8]. Importantly, fish-oil-based emulsions rich in omega-3 fatty acids have demonstrated the ability to reverse PN-associated cholestasis [9]. However, eliminating omega-6 fatty acids is not recommended due to the risk of essential fatty acid deficiency, particularly in neonates [2]. Current strategies, therefore, focus on improving the omega-6/omega-3 ratio while ensuring the supply of both fatty acid classes [2,10,11].

Beyond modifying fatty acid composition and reducing phytosterol load, enriching PN emulsions with hepatoprotective compounds represents a promising strategy to mitigate IFALD.

Many compounds have been documented to have hepatoprotective effects. One of the most commonly described is silymarin, which is obtained from *Silybum marianum*. It is a complex of flavonolignans with anti-inflammatory and immunomodulatory properties [12]. Their mechanism of action involves stabilizing hepatocyte cell membranes and reducing liver enzyme activity, thereby limiting oxidative damage and lipid peroxidation. These compounds activate antioxidant pathways, including Nrf2, thereby reducing oxidative stress. Additionally, silymarin lowers cholesterol, triglyceride, and LDL concentrations. It also affects the expression of genes related to lipogenesis, including SREBP-1c, LXRα, and FAS. Furthermore, it influences the secretion of pro-inflammatory cytokines, such as TNF-α and IL-6, and modulates the NF-κB and NLRP3 pathways [13]. Silymarin is used to treat various liver diseases, including non-alcoholic fatty liver disease, alcoholic liver disease, hepatic cancers, and non-alcoholic steatohepatitis [12].

Another widely used compound is ursodeoxycholic acid (UDCA), which primarily acts by displacing toxic hydrophobic bile acids from cell membranes and stabilizing hepatocyte membranes [14,15]. UDCA also has antioxidant, immunomodulatory, and anti-apoptotic properties, and its effectiveness has been confirmed in cholestatic liver diseases and models of non-alcoholic steatohepatitis, among others [16].

In the context of liver protection, compounds that increase the availability of intracellular thiol groups have also been studied. These include 4-thiazolidinecarboxylic acid derivatives, which act as cysteine prodrugs to increase glutathione synthesis and support hepatocyte detoxification processes [17].

**Figure 1:**
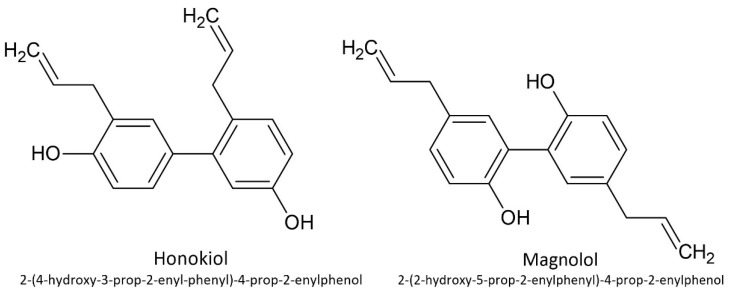
Chemical structures of MAG and HON.

In recent years, there has been growing interest in natural bioactive compounds with multidirectional effects, such as polyphenols and plant lignans. Honokiol (HON) and magnolol (MAG), two neolignans found in *Magnolia officinalis* and *Magnolia obovata*, are desirable candidates. The structure of these positional isomers contains a biphenyl group are presented in Figure 1. They possess strong antioxidant, anti-inflammatory, anti-necroptotic, and cytoprotective properties and have demonstrated hepatoprotective effects across multiple preclinical models [18,19,20,21,22,23,24].

MAG reduces ALT and AST levels, alleviates oxidative stress, and suppresses inflammatory pathways in alcoholic liver disease [18]. HON exhibits similar protective effects by modulating inflammatory cytokines and lipid metabolism [19]. MAG also protects against acetaminophen-induced hepatotoxicity by reducing lipid peroxidation and restoring glutathione levels [20]. In metabolic fatty liver disease, HON reduces lipid accumulation and oxidative stress by activating Nrf2 and modulating RIPK3 signaling, thereby slowing disease progression [21]. In NAFLD, HON activates AMPK and directly binds its γ1 subunit, reducing steatosis and hepatic inflammation [22]. Both HON and MAG protect hepatocytes from tert-butylhydroperoxide and D-galactosamine toxicity, reducing lipid peroxidation and preserving membrane integrity [23]. Additionally, HON modulates RXR-mediated signaling [24], while MAG interacts with LXRα [19]. These nuclear receptors play key roles in lipid and cholesterol metabolism, which are critical processes involved in PN-associated liver dysfunction (IFALD) [25]. Thus, the ability of HON and MAG to modulate these receptors distinguishes them from other hepatoprotective agents and highlights a novel therapeutic approach for influencing the course of IFALD.

Given their lipophilicity, HON and MAG require a suitable delivery system for intravenous use. Nanoemulsions are particularly advantageous because they enable the incorporation of hydrophobic bioactives into a biocompatible lipid matrix while preserving the physicochemical requirements for intravenous administration. Their small droplet size and high kinetic stability make them suitable carriers for combining nutritional and therapeutic functions.

Based on these considerations, this study aimed to develop novel neolignan-loaded nanoemulsions and to evaluate their physicochemical stability, stress resistance, sterility, biocompatibility, and compatibility with commercial PN admixtures. The overarching goal was to create a pharmacopeia-compliant lipid system with added hepatoprotective functionality that may support the prevention and management of IFALD.

## 2. Results

### 2.1. The Optimization of the High-Pressure Homogenization Process

**Figure 2:**
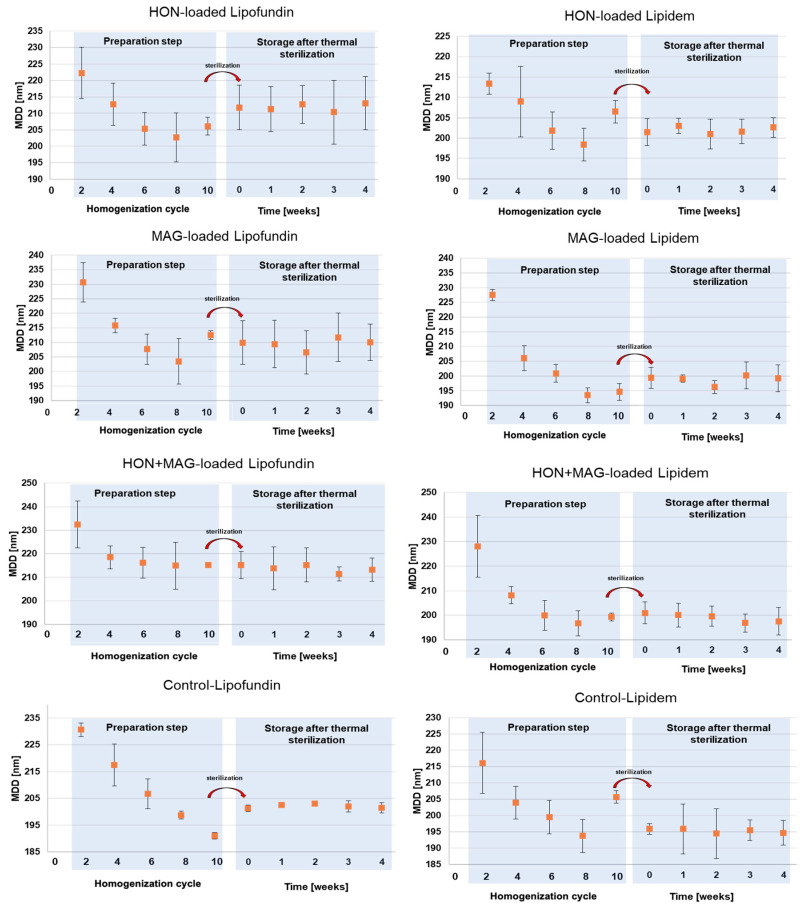
MDD results of the studied nanoemulsions during homogenization optimization and mid-term stability studies.

The optimization stage evaluated the impact of high-pressure homogenization cycles on the physicochemical properties of the neolignan-loaded nanoemulsions. Increasing the number of cycles led to a progressive reduction in both MDD and PDI, indicating gradual refinement of the dispersed phase (Figure 2). The most pronounced decrease in MDD occurred between the second and eighth homogenization cycles and was statistically significant in all formulations. Increasing the number of high-pressure homogenization cycles from 8 to 10 did not substantially change the physicochemical properties of the formulations (except Control-Lipofundin). Therefore, further increasing the number of cycles was deemed unnecessary, and the homogenization process was finalized at 10 cycles.

### 2.2. Physicochemical Characterization and Mid-Term Stability Evaluation

Following optimization, the physicochemical properties of nanoemulsions were evaluated and compared with pharmacopeial requirements for intravenous lipid formulations. Immediately after preparation and thermal sterilization (t = 0), all samples exhibited a uniform and homogeneous appearance without visible signs of phase separation or particulate matter. MDD values ranged from 195 to 215 nm, remaining well below the commonly accepted upper size threshold of approximately 500 nm for injectable formulations [26], and PDI values (0.036–0.076) confirmed narrow droplet size distribution and high system homogeneity. The ZP values were strongly negative (−41.7 to −49.5 mV), indicating sufficient electrostatic repulsion to prevent droplet coalescence. The pH values (7.56–7.96) and osmolality (340–358 mOsm/kg H_2_O) of these formulations remained within the acceptable range for intravenous administration. These formulations generally exhibit a pH between 6 and 9, and the acceptable osmolality varies by administration route. Limits are approximately 1000 mOsm/kg for peripheral veins and up to 3000 mOsm/kg for central venous administration [27]. Importantly, PFAT5 remained below the critical limit of 0.05% in all samples, confirming the absence of large, clinically hazardous droplets. Only minor, insignificant differences in tested parameters were observed between HON-, MAG-, HON+MAG-loaded, and unloaded nanoemulsions.

After four weeks of refrigerated storage, the nanoemulsions retained their physicochemical stability. Changes in MDD were minimal, ranging from an increase of 1.3 nm (HON-loaded Lipofundin) to a decrease of 3.4 nm (HON+MAG-loaded Lipofundin), indicating no detectable droplet growth or destabilization (Figure 2). PDI values remained within the initial range (0.036–0.076), with no statistically significant differences observed in any of the formulations. A statistically significant increase in ZP was detected only in the magnolol-containing nanoemulsions, where values reached up to −48.7 mV, indicating improved interfacial stabilization over time. Variations in other formulations were not statistically significant. The PFAT5 parameter remained below 0.05% across all samples throughout storage, demonstrating the continued absence of large droplets and confirming suitability for intravenous administration. Both pH and OSM values exhibited only slight fluctuations, remaining within physiologically and technologically acceptable ranges. Chemical stability was also preserved. After four weeks, only minor decreases of 1–3% were observed in some Lipofundin-based samples (HON-loaded, MAG-loaded, and HON+MAG-loaded), although these changes remained well within the ±10% pharmacopoeial threshold for active substance content [28]. The detailed results are presented in Table 1.

### 2.3. Stress-Response Evaluation

The stress-response studies evaluated the behavior of the developed nanoemulsions under destabilizing conditions, including acidic and alkaline environments, oxidative stress, elevated temperature, and repeated freeze–thaw cycling (Tables S1–S3). These experiments provided insight into the robustness of the formulations and the influence of the incorporated lignans on their physicochemical stability.

Exposure to acidic conditions resulted in immediate destabilization of all tested nanoemulsions (Table S1). A rapid increase in MDD and PDI values, accompanied by ZP approaching zero, indicated loss of electrostatic stabilization and the onset of extensive droplet coalescence. Complete phase separation occurred early in the experiment, preventing further measurements beyond t = 0, and confirming that the formulations are highly sensitive to acidic environments, irrespective of the lignan type or lipid matrix. Under alkaline stress, all formulations initially remained stable, maintaining acceptable MDD, PDI, and PFAT5 values during the first 48 h. After seven days, however, clear differences emerged. Formulations containing HON alone and the unloaded control emulsions showed signs of coagulation and structural breakdown, whereas MAG-loaded and HON+MAG-loaded nanoemulsions maintained their stability. These results suggest that MAG provides a stabilizing effect in alkaline conditions, while HON increases susceptibility to destabilization. Notably, the combination of both lignans mitigated the destabilizing influence of HON, indicating complementary interactions at the droplet interface. Oxidative stress did not induce significant physicochemical changes in any of the tested formulations. No phase separation was observed, and MDD and PDI remained stable throughout the experiment. PFAT5 values consistently remained below the critical threshold, and encapsulation efficiency varied by no more than ±4% compared with initial measurements. These results highlight the high resistance of the developed nanoemulsions to oxidative degradation and confirm the chemical stability of the encapsulated lignans under oxidative conditions.

Thermal stress at 60 °C led to gradual but pronounced destabilization. In neolingan-loaded Lipofundin-based nanoemulsions, PFAT5 values exceeded the pharmacopoeial threshold after 24 h, while in Lipidem-based emulsions this occurred after 48 h. In contrast, unloaded control nanoemulsions demonstrated markedly greater thermal resilience, maintaining acceptable PFAT5 levels for up to 72 h. Although the neolignan-loaded samples displayed increased PFAT5 values under heat exposure, encapsulation efficiency remained high at early time points. At later stages, however, extensive droplet growth and phase separation prevented further quantitative assessment. These findings indicate that, while HON and MAG remain chemically stable at elevated temperatures, their presence reduces the physical stability of the nanoemulsions during prolonged thermal stress.

Freeze–thaw cycling provided the most discriminative assessment of formulation robustness. The results of PFAT5 and EE% after the freeze–thaw cycles are presented in Table 2. While encapsulation efficiency remained high across all formulations, with only slight 1–4% reductions in some HON-loaded samples, PFAT5 analysis revealed substantial differences in physical stability. HON-loaded nanoemulsions exceeded the PFAT5 limit after the second and third cycle, indicating significant droplet coalescence. Emulsions containing both neolignans (HON+MAG) destabilized even more rapidly, exceeding the critical PFAT5 threshold after the first cycle, after which complete phase separation prevented further measurements. In contrast, MAG-loaded nanoemulsions and unloaded controls remained fully stable throughout all three freeze–thaw cycles, maintaining PFAT5 values within acceptable limits. These outcomes indicate that HON adversely affects freeze–thaw stability, whereas MAG has a protective effect that helps preserve nanoemulsion structure under severe temperature fluctuations.

Although HON and MAG are structural isomers with similar lipophilic properties (log P ≈ 4.5), it should be emphasized that they may exhibit different physicochemical properties. According to Usach et al. [29], HON’s stability decreases with increasing pH due to its greater susceptibility to degradation in an alkaline environment. In our study, incorporating HON in a nanoemulsion limited its degradation. However, the formulation itself exhibited lower physical stability under alkaline conditions and during freeze–thaw cycles compared to the nanoemulsion containing MAG. The results suggest that MAG may stabilize nanoemulsions exposed to different pH conditions and freeze–thaw cycles more effectively. This effect may be related to the position of the hydroxyl groups on the phenyl ring, which differs from that of HON, and their ability to form hydrogen bonds. The relationship between the chemical structure of these neolignans and their functional properties, with regard to antioxidant activity, has already been described in the literature. Amort et al. [30] demonstrated that the observed differences stem from the formation of intramolecular hydrogen bonds between hydroxyl groups, as well as from their interaction with aromatic and allylic systems.

Taken together, the stress-response studies demonstrate that the developed nanoemulsions exhibit robust stability under oxidative and moderate alkaline conditions, while remaining sensitive to acidic environments, prolonged heating, and repeated freeze–thaw cycling.

MAG consistently enhanced physical stability across several stress conditions, whereas HON reduced stability, particularly under thermal and freeze–thaw stress. The combination of both lignans provided partial mitigation in some cases, reflecting complex interfacial interactions between the compounds and the lipid matrix.

### 2.4. Evaluation of Sterility

Sterility is a critical requirement for all intravenous formulations, as the presence of microorganisms may pose a direct risk to patient safety and compromise the stability of the preparation. Following literature guidelines, thermal sterilization using saturated steam at 121 °C for 20 min was selected as the most appropriate method for the developed nanoemulsions. After autoclaving, the sterility of the samples was confirmed, demonstrating the effectiveness of the applied procedure. Physicochemical evaluation revealed that the sterilization process did not negatively affect the quality of the nanoemulsions. Only a slight increase in MDD and a decrease in pH values were observed, while PDI, ZP, PFAT5, and OSM remained almost unchanged. The results confirm that the applied method is suitable for obtaining a stable sterile formulation.

### 2.5. In Vitro Cytotoxicity

**Figure 3:**
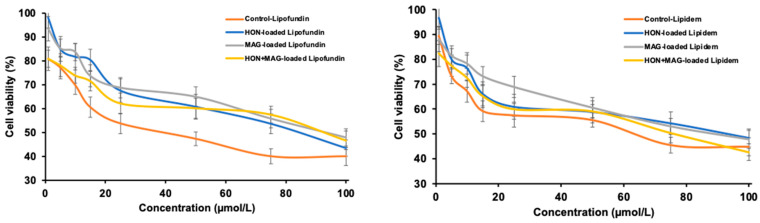
Effect of HON-, MAG-, HON+MAG-loaded nanoemulsions on THLE-2 cell viability after 24 h incubation, assessed by the MTT assay. Data are presented as mean ± SEM (*n* = 3).

The cytotoxicity of the developed nanoemulsions was assessed using the MTT assay in THLE-2 human liver epithelial cells (Figure 3). Cells were exposed for 24 h to neolignan-loaded nanoemulsions at final concentrations of HON or MAG ranging from 1 to 100 μM. Both Lipofundin- and Lipidem-based nanoemulsions showed a clear dose-dependent decrease in THLE-2 cell viability across the 1–100 µM range. In each case, neolignan-loaded formulations (HON, MAG, HON+MAG) exhibited higher cell viability than their unloaded controls, indicating reduced cytotoxicity upon neolignan incorporation. The statistical significance of the results is presented in Supplementary Figure S1. Among all tested formulations, MAG-loaded nanoemulsions consistently demonstrated the highest cell survival, suggesting the most favorable safety profile.

### 2.6. Hemolysis Test

Assessment of hemolytic activity was conducted to evaluate the biocompatibility of the formulations with RBC and confirm their suitability for intravenous administration. Each nanoemulsion was incubated with human erythrocytes, and hemolysis was quantified relative to positive and negative controls. All tested formulations exhibited very low hemolytic potential, with values ranging from 0% to 2.11%, remaining well below the accepted safety threshold of 5% (Table 3). Neither the type of lipid matrix (Lipofundin vs. Lipidem) nor the presence of HON, MAG, or their combination resulted in excessive erythrocyte damage. These results demonstrate a favorable hemocompatibility profile and confirm that the developed nanoemulsions are safe for intravenous use in terms of RBC integrity.

### 2.7. Compatibility with Parenteral Nutrition Admixtures

**Figure 4:**
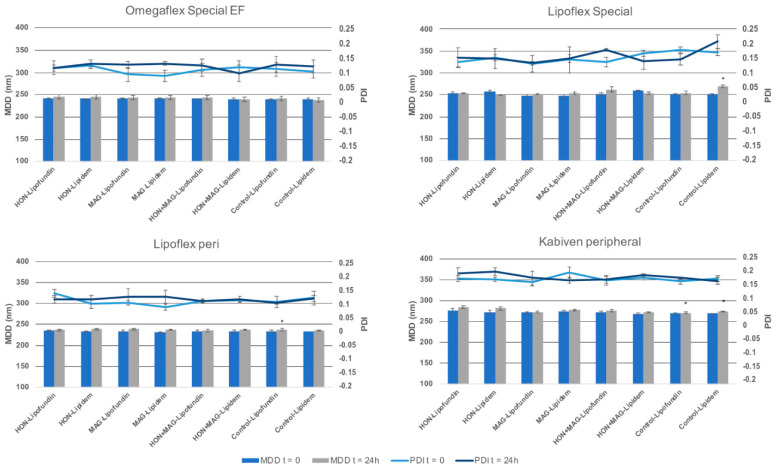
The results of mean droplet diameter (MDD) and polydispersity index (PDI) at t = 0 and after 24 h in PN admixtures, combined with the developed nanoemulsions. Data are presented as mean ± SD. * Statistically significant differences (*p* < 0.05).

The compatibility between the developed nanoemulsions and commercially available PN admixtures was evaluated to determine their potential clinical applicability. Four commercial PN admixtures were selected for comprehensive assessment: LSpec, OSpec, Lperi, and KPeri. After mixing nanoemulsions with the PN admixtures, no phase separation or color changes were observed immediately and after 24 h of storage. MDD and PDI values remained within acceptable limits across all mixtures, with MDD ranging from 230.9 to 285.2 nm and PDI from 0.088 to 0.205 (Figure 4). ZP values (−37.1 to −18.2 mV) confirmed adequate colloidal stability. PFAT5 remained below the USP limit of 0.05% in all tested combinations, both at t = 0 and after 24 h (Table 4). In many cases, PFAT5 values decreased over time, except for slight increases observed in formulations combined with LSpec, but still within the acceptance limits. Statistical analysis revealed significant differences in MDD between t = 0 and t = 24 h only for admixtures containing unloaded emulsions, while lignan-loaded formulations showed no destabilization. PDI values remained stable, and significant changes in ZP were limited to only two combinations (HON-loaded Lipofundin with OSpec and HON-loaded Lipofundin with KPeri). pH and OSM values exhibited only minor deviations from the original PN formulations and remained within physiologically acceptable ranges (Table 5). Collectively, these findings indicate that the developed nanoemulsions are compatible with the tested PN admixtures and do not compromise their physicochemical stability, supporting their potential use as carriers of bioactive compounds within PN.

## 3. Discussion

HON and MAG, two major neolignans derived from *Magnolia* spp., have attracted sustained scientific interest due to their broad spectrum of biological activities, including antioxidant, anti-inflammatory, and hepatoprotective effects. Their ability to modulate oxidative stress and inflammatory pathways makes them particularly relevant in conditions associated with liver dysfunction, such as parenteral nutrition–associated complications [31]. However, their pronounced lipophilicity limits aqueous solubility and restricts their clinical translation, especially for intravenous administration [29]. Nanoemulsions offer a promising strategy to overcome these challenges by enabling the incorporation of hydrophobic compounds within a biocompatible lipid matrix, improving dispersibility, and protecting the drug from degradation [32]. Thus, HON and MAG serve not only as pharmacologically attractive molecules but also as rigorous model compounds for assessing the performance of lipid-based intravenous delivery systems.

This relevance becomes especially apparent in the context of PN, where lipid emulsions play a central role as both energy sources and structural carriers. Lipofundin and Lipidem, two clinically established nanoemulsions, exemplify the physicochemical performance required for safe intravenous administration, including small and uniform droplet size, high electrostatic stabilization, and minimal formation of large droplets. Their lipid compositions differ in physiological functionality: Lipofundin contains a mixture of MCT and long-chain triglycerides (LCT), whereas Lipidem additionally incorporates omega-3 triglycerides, which provide immunomodulatory benefits [33]. Mixing Lipofundin in a 1:1 ratio with the neolingnan-loaded pre-emulsions containing MCT and fish oil in the volume ratio of 1:1 results in a change in the proportion of soybean-derived ω-6 fatty acids and fish-oil-derived ω-3 fatty acids, yielding an approximate ω-6:ω-3 ratio of 1:1. In the case of for Lipidem, whose lipid phase contains ~40% soybean oil, 50% MCT and 10% fish oil, a 1:1 mixture with the same pre-emulsions gives the ω-6:ω-3 balance shifts from roughly 4:1 in the original emulsion to about 0.7:1 in the mixed system, further enriching the formulation in ω-3 fatty acids. Table 6 summarizes the changes in lipid-phase composition resulting from mixing the MCT/fish-oil pre-emulsion with Lipofundin or Lipidem. Preparing nanoemulsions by mixing neolignan-loaded pre-emulsions with Lipofundin or Lipidem enables the generation of distinct fatty acid profiles. This approach not only allows assessment of neolignan effects on stability and biocompatibility, but also produces formulations with a markedly increased content of omega-3 fatty acids and a reduced proportion of omega-6 fatty acids, thereby permitting evaluation of the impact of lipid composition on the overall performance of the emulsions.

MDD between 195 and 215 nm, narrow size distributions (PDI 0.036–0.076), and strongly negative ZP (−41.7 to −49.5 mV). These values indicate high colloidal stability and effective droplet repulsion, consistent with the performance expected from clinically approved emulsions. Importantly, the incorporation of HON and MAG did not compromise droplet size, homogeneity, or surface charge, demonstrating that both neolignans can be successfully integrated into the lipid matrix without disrupting fundamental structural properties.

The nanoemulsions also maintained excellent stability over four weeks of storage. Variations in MDD were minimal (<3 nm), PDI values remained within the initial narrow range, and PFAT5 values consistently stayed below the critical 0.05% threshold. These findings confirm mid-term physical stability and the absence of clinically hazardous large droplets. MAG-containing formulations even exhibited a slight increase in the magnitude of the ZP, suggesting progressive reinforcement of the interfacial layer. In addition to standard physicochemical characterization, short-term stress tests were performed. Although these conditions exceed typical clinical storage and handling scenarios, they provide a valuable assessment of the structural stability of emulsions under extreme conditions and the role of lignans in modulating this stability. Nevertheless, from a practical point of view, the physicochemical properties of nanoemulsions subjected to thermal stress may be relevant in PN practice. Parenteral nutrition admixtures are administered at ambient temperature, where strict temperature control is not always possible. Therefore, the results obtained under elevated temperature conditions may reflect real-world handling variability in PN practice to some extent. However, HON exerted a destabilizing effect only under severe freeze–thaw stress, while under standard storage conditions, all HON-containing emulsions remained fully stable.

From a technological standpoint, the method used to produce these nanoemulsions, high-pressure homogenization, offers significant advantages for scalability and potential clinical translation [32]. The process is widely applied in the pharmaceutical and food industries, and the nanoemulsion characteristics observed in this study were achieved using a straightforward and reproducible ten-cycle protocol. No specialized excipients or complex multi-step procedures were required, underscoring the feasibility of adapting this method for industrial-scale and GMP-compliant production.

Only a limited number of studies have explored intravenous nanoemulsions containing studied neolignans [32,34,35], highlighting the innovative nature of the present work and its relevance for improving the safety of long-term PN. Overall, the findings indicate that HON- and MAG-loaded nanoemulsions combine the physicochemical robustness of established PN nanoemulsions with the added therapeutic potential of delivering hepatoprotective compounds. Their stability, compatibility with commercial PN admixtures, and scalable manufacturing process support their feasibility as next-generation functional nanoemulsions for parenteral nutrition, particularly in the prevention and management of IFALD.

## 4. Materials and Methods

### 4.1. Materials

MAG and HON were purchased from Pol-Aura, Olsztyn, Poland. Purified fish oil (Ph. Eur. Type I), MCT (Ph. Eur., USP), egg yolk lecithin, and sodium oleate were kindly gifted by Lipoid GmbH (Ludwigshafen, Germany). Lipofundin MCT/LCT 20% (Lipofundin), Lipidem, Water for injection, Lipoflex Special (LSpec), Omegaflex Special EF (OSpec), Lipoflex Peri (Lperi), and Tracutil were purchased from B. Braun Melsungen AG, Melsungen, Germany. Kabiven Peripheral (KPeri) was purchased from Fresenius Kabi AB, Uppsala, Sweden. Cernevit was purchased from Baxter, Warsaw, Poland. All organic solvents used in the studies were of analytical or high-performance liquid chromatographic grade. Epithelial human liver cells, THLE-2 (CRL-2706), were purchased from the American Type Culture Collection (ATCC), Manassas, VA, USA. Human red blood cells (RBCs) were purchased from the Regional Blood Donor Center in Poznan, Poland.

### 4.2. Preparation of Neolignan-Loaded Nanoemulsion

Neolignan-loaded nanoemulsions were prepared using a two-step high-energy emulsification process involving high-shear homogenization followed by high-pressure homogenization. The oil and aqueous phases were prepared separately. The oil phase consisted of 20 g of a 1:1 (*w*/*w*) mixture of medium-chain triglycerides (MCT) and fish oil. HON and MAG were dissolved directly in the oil phase. For single-neolignan formulations, 2.8 g of either HON or MAG was incorporated into the 20 g oil phase, whereas dual-neolignan formulations contained 1.4 g HON and 1.4 g MAG. The oil mixture was heated to 40 °C and stirred until complete dissolution of the neolignans for 15 min. The aqueous phase was prepared by dissolving egg-yolk lecithin, sodium oleate, and glycerol in water for injection under continuous stirring (500 rpm) at 70 °C until complete dissolution was achieved. After preparing both phases, the aqueous phase was subjected to high-shear homogenization for 10 min at 17,000 rpm. During homogenization, the oil phase was added dropwise to facilitate the formation of a coarse pre-emulsion.

Immediately after preparation, the resulting pre-emulsion was cooled on ice, then mixed with commercial lipid emulsions (Lipofundin or Lipidem) at a 1:1 volume ratio, gently stirred to ensure uniform distribution, and subject to a high-pressure homogenization (GEA PandaPLUS 2000, GEA Niro Soavi, Düsseldorf, Germany) at 800 bar. During homogenization, the emulsions were cooled in an ice-water bath to maintain thermal stability and prevent heat-induced degradation of formulation components. The optimization of the high-pressure homogenization process focused on determining how the number of homogenization cycles influenced the physicochemical properties of the nanoemulsions. Pre-emulsions were homogenized at 800 bar, and 1 mL aliquots were collected after every two homogenization cycles. The impact of the progressive number of cycles on mean droplet diameter (MDD) and polydispersity index (PDI) was assessed.

After homogenization, the pH of each formulation was adjusted to 8.5 with 0.5 mol/L NaOH. Then, samples were transferred into 20 mL injection vials, closed under a nitrogen atmosphere to prevent oxidative degradation, and sterilized. Thermal sterilization was carried out in a steam autoclave at 121 °C for 20 min. Following processing, the nanoemulsions were stored at 4 ± 1 °C and protected from light. Formulations containing HON, MAG, and the combination of HON and MAG, as well as neolignan-free controls, were prepared in triplicate to ensure reproducibility. The detailed quantitative composition of the developed nanoemulsion is provided in Table 7.

### 4.3. Physicochemical Properties of Nanoemulsions

Physicochemical characterization of the neolignan-loaded nanoemulsions included assessment of MDD, PDI, ZP, pH, osmolarity (OSM), and PFAT5. MDD, PDI, and ZP were measured using a Zetasizer Nano ZS (Malvern Instruments, Worcester, UK), which employs dynamic light scattering (DLS) and laser Doppler electrophoresis methods. The instrument uses a 633 nm laser operating at a 173° backscatter angle, and all measurements were performed at 25 °C. Prior to analysis, the samples were diluted 1:100 with water for injection. pH measurements were conducted using a SevenCompact pH meter (Mettler Toledo, Columbus, OH, USA), and the OSM was assessed using the freezing point depression method with an Osmometer 800 CLG (Tridentmed, Warsaw, Poland). Both instruments were calibrated immediately before use according to the manufacturer’s instructions. The PFAT5 was determined using a PAMAS SVSS particle counter (Partikelmess- und Analysesysteme GmbH, Rutesheim, Germany), which employs light obscuration with a single-particle optical sizing technique of individual particles. Before analysis, samples were diluted 1:2000 with water for injection, and PFAT5 values were calculated according to the method described by Peng et al. [36]. Each parameter was determined in triplicate.

### 4.4. Lignans Quantification and Entrapment Efficiency Assessment

MAG and HON concentrations were determined using high-performance liquid chromatography (HPLC). The analysis was carried out on an Agilent 1260 Infinity II LC system (Agilent Technologies, Waldbronn, Germany) equipped with a DAD detector. Detection wavelengths were set at 290 nm for MAG and 255 nm for HON. A reversed-phase C18 column (Luna 100 Å, 150 × 4.6 mm i.d., 5 µm; Phenomenex, Torrance, CA, USA) was used for separation. The mobile phase consisted of 0.4% acetic acid (21%), acetonitrile (25%), and methanol (54%) [32]. The total run time was 10 min, and the injection volume was 10 µL. Before analysis, samples were prepared by mixing a 100 µL aliquot of the nanoemulsion containing MAG, HON, or their combination with 1 mL of dichloromethane and diluting the mixture to 10 mL with methanol. The resulting solution was injected onto the chromatography column. The calibration curve for HON was prepared in accordance with the method described by Dominiak et al. [34], and was linear over the range of 0.005–0.16 μg/mL (y = 24,937x ± 4.0139; r = 0.9999). The calibration curve for MAG was linear over the range of 0.0150–0.3 mg/mL (y = 14,983x ± 46.952; r = 0.9997). The limits of detection (LOD) and quantitation (LOQ) for MAG were 0.0044 mg/mL and 0.0134 mg/mL, respectively; for HON, they were 0.00147 mg/mL and 0.0044 mg/mL, respectively.

Encapsulation efficiency (EE%) was calculated according to the following equation:

(1) $$EE\%=\frac{Quantified\;concentration\;of\;lignan}{Theoretical\;concentration\;of\;lignan}\times 100\%$$

### 4.5. Integrated Physicochemical Stability and Stress-Response Evaluation

Integrated physicochemical stability and stress-response studies were conducted to assess the impact of various external stress factors on the behavior of lignan-loaded nanoemulsions. Although these conditions do not conform to the standard for storing preparations, they enable the stability of nanoemulsions to be evaluated within a shorter timeframe.

Firstly, the mid-term stability was assessed by monitoring physicochemical parameters (MDD, PDI, ZP, pH, OSM, PFAT5, and EE%) at predefined time points (0, 7, 14, 21, and 28 days) during storage at 4 ± 1 °C under light-protected conditions.

Then, short-term stress testing was performed to assess the nanoemulsions’ response to chemical and thermal challenges, following the methodology described by Gostyńska et al. [32]. For stress testing, 3 mL of each nanoemulsion was mixed with an equal volume of 0.5 M NaOH, 0.5 M HCl, or 30% H_2_O_2_ to induce alkaline, acidic, or oxidative stress, respectively. The samples were then stored at 25 ± 1 °C without light exposure. Thermal stress was assessed separately by incubating the nanoemulsions in a thermostatic chamber at 60 ± 1 °C. At predetermined intervals (0, 24, 48, and 72 h), physicochemical parameters (MDD, PDI, ZP, pH, OSM, and PFAT5) were analyzed, and lignan content was quantified by HPLC and expressed as EE%.

Finally, freeze–thaw stability was subsequently assessed using a Crystal 16 multi-reactor crystallizer (Avantium Technologies, Amsterdam, The Netherlands). The nanoemulsions underwent three controlled freeze–thaw cycles between −5 ± 1 °C and 40 ± 1 °C, with a temperature ramp of 1 °C/min and 20 min holds at each extreme. The impact of repeated freeze–thaw cycles was assessed by analyzing MDD, PDI, ZP, and PFAT5, whereas changes in neolignan retention (EE%) were quantified by HPLC. All experiments were performed in three independent replicates.

### 4.6. Assessment of the Sterility

The sterility test was performed using the direct inoculation method, as specified in the European Pharmacopeia. Briefly, the tested nanoemulsions were added to liquid thioglycollate medium (TG) and Tryptone Soya Broth (TSB) (Millipore S.A.S., 67120 Molsheim, France), with the volume of the substance accounting for 10% of the medium volume. The media were incubated for 14 days at 34 °C (TG) and 22 °C (TSB). After this period, 1 mL was transferred to fresh media accordingly and incubated again for 4 days at the same temperatures. After incubation, microbial growth was observed; the absence of growth indicated that the tested product met sterility requirements.

### 4.7. In Vitro Cytotoxicity Studies

The viability of human epithelial liver THLE-2 cells exposed to neolignan-loaded nanoemulsions was assessed using the MTT assay. The results were compared with those obtained for lignan-free controls. For this assay, human epithelial liver cells THLE-2 were cultured in bronchial epithelial growth medium (BEGM) supplemented with the BEGM Bullet Kit (Lonza, Cologne, Germany), 10% fetal bovine serum (FBS, EURx, Gdańsk, Poland), 5 ng/mL epidermal growth factor (EGF) and 70 ng/mL phosphoethanolamine (Sigma-Aldrich, St. Louis, MO, USA). Cells were maintained at 37 °C in a humidified atmosphere containing 5% CO_2_. THLE-2 cells were seeded into 96-well plates at a density of 1 × 10^4^ cells per well and pre-incubated for 24 h in complete medium. Diluted emulsions were then added at final neolignan concentrations ranging from 1 to 100 μM, followed by a 24 h incubation period. After exposure, cells were washed with phosphate-buffered saline (PBS), and fresh complete medium containing MTT reagent (0.5 mg/mL) was added. Cells were incubated for an additional 4 h to allow for formazan formation. The resulting crystals were dissolved in isopropanol containing hydrochloric acid, and absorbance was measured at 750 nm with background correction at 690 nm using a Infinite M200 microplate reader (TECAN, Grödig, Austria).

Statistical analyses were performed using GraphPad InStat 3 software.

### 4.8. Hemolysis Assay

To assess the hemolytic potential of the neolignan-loaded nanoemulsions, their interaction with human red blood cells (RBC) was examined. To obtain an RBC suspension with a physiological adult hematocrit, 6 mL of pediatric RBC concentrate was diluted with 2 mL of PBS. The tested nanoemulsions were then added at a 1:100 (*v*/*v*) ratio and incubated for 60 min at 37 °C under continuous shaking. Following incubation, the mixtures were centrifuged at 1200 rpm for 10 min, and 2 mL of the resulting supernatant was transferred to EDTA monovettes for analysis. An untreated RBC suspension processed under identical conditions served as a baseline control, while Triton X-100 and 0.9% NaCl acted as positive and negative controls, respectively. The XN-10 automatic hematology analyzer was used to measure hemoglobin concentration in the collected supernatant. The device is approved by the US Food and Drug Administration (FDA) and operates on the basis of fluorescence flow cytometry (FFC) using a semiconductor laser. The hemoglobin measurement method employs cyanomethemoglobin and sodium lauryl sulphate (SLS) without cyanide, which induces lysis of red and white blood cells. This leads to a chemical reaction involving the conversion of globin and the oxidation of the hem group. The hydrophilic groups of the SLS molecules bind to the hem group to form a stable, colored complex (SLS-HGB), which is analyzed by photometry. The LED emits monochromatic light that is absorbed by the SLS-HGB complexes when passing through the mixture. The absorbance measured by the photoelectric sensor is proportional to the hemoglobin concentration in the sample.

Hemolysis (%) was calculated using the equation:

(2) $$Hemolysis=\frac{C_{sample}-C_{negative}}{C_{positive}-C_{negative}},$$

where C_negative_, C_positive_, and C_sample_ are the hemoglobin concentrations of the negative control, positive control, and tested samples, respectively.

### 4.9. Compatibility of Nanoemulsions with Parenteral Nutrition Admixtures

Compatibility studies were performed to assess the suitability of incorporating the developed neolignane-loaded nanoemulsions into PN admixtures. The quality and stability of the supplemented preparations were evaluated over 24 h, simulating the maximum clinical infusion time of PN.

Because no clinical data exist on intravenous dosing of HON and MAG in humans, a preliminary dose estimation was required. This estimation was based on extrapolating doses reported in animal studies available in the literature. To achieve this, the coefficient-based method described by Nair et al. [37], which is recommended by the FDA for human equivalent dose (HED) determination, was applied. This empirical approach reduces the risk of toxicity and is preferred over simple body-weight scaling, as it accounts for interspecies differences in metabolic rate and physiology. The resulting HED should be considered indicative only and not as the maximum recommended starting dose (MRSD) for clinical trials. The calculation used the intravenous dose of HON/MAG administered to rats (20 mg/kg b.w.) in the studies by Lin [38] and Lin et al. [39], according to the following equation:

(3) $$HED=Animal\;dose\;(\frac{mg}{kg})\times (\frac{Animal\;Km}{Human\;Km})$$

where HED is the human equivalent dose, and Km is the body surface area normalization factor, which takes the value of 6 for rats and 37 for humans.

The calculated HED for HON and MAG was 3.24 mg/kg. Assuming an average neolignan concentration of 9 mg/mL in the nanoemulsion, this would correspond to approximately 25.2 mL of nanoemulsion for a 70 kg adult per 2500 mL of PN. For safety reasons, the administered volume was reduced to 10 mL in the experiments, which provided a total neolignan dose of about 90 mg and was added to 1250 mL of PN admixture, corresponding to the standard 24 h infusion volume.

Compatibility was determined with the following commercial PN admixtures: LSpec (Lipoflex Special), OSpec (Omegaflex Special), Lperi (Lipoflex Peri), and KPeri (Kabiven Peripheral). Before adding the tested nanoemulsions, the PN mixtures were supplemented with vitamins (Cernevit) and trace elements (Tracutil). Compatibility was assessed based on visual analysis and measurements of MDD, PDI, ZP, pH, OSM, and PFAT5. The parameters were determined immediately after the samples preparation and after 24 h of storage at 20 ± 2 °C. Samples were prepared in duplicate.

### 4.10. Statistical Analysis

Data are presented as mean ± standard deviation (SD). Experiments were conducted in duplicate or triplicate. Comparisons between two groups were analyzed using a two-tailed Student’s *t*-test, whereas one-way ANOVA was used for comparisons among three or more groups. A *p*-value less than 0.05 was considered statistically significant. All statistical analyses were carried out using GraphPad InStat 3 or Microsoft Excel software.

## 5. Limitations and Perspectives

The present study has several limitations that should be taken into consideration. First, the stability assessment of the developed nanoemulsions was performed over a four-week period. Although this timeframe is sufficient for preliminary evaluation, mid-term stability studies in the literature often extend up to 180 days. Future research should therefore include additional time points and longer observation periods to more comprehensively evaluate physicochemical stability. Likewise, the influence of different environmental conditions, such as long-term storage at room temperature or exposure to light, should be assessed, as these factors may further affect nanoemulsion integrity.

Second, the biological evaluation was limited to in vitro testing on THLE-2 hepatocyte cells. While these experiments provide useful preliminary insights into cytotoxicity and cellular responses, in vivo studies will be necessary to assess the systemic safety, biodistribution, and therapeutic potential of HON- and MAG-loaded nanoemulsions. Given that the formulations meet all relevant pharmacopeial requirements, such studies are feasible and could provide valuable data on the effects of neolignans and increased omega-3 content on physiological and biochemical parameters in animal models.

Finally, an essential consideration for future translational studies is the establishment of a safe intravenous dose for HON and MAG. For these lignans, human intravenous dosing data are currently unavailable, and the evidence is limited to animal studies. Therefore, determining an appropriate starting dose for humans will be necessary before initiating clinical trials. In this study, we used the HED estimation method proposed by Nair et al. [37] to guide dose calculation, providing a solid framework for subsequent in vivo and clinical investigations.

## 6. Conclusions

In summary, this study presents a robust and scalable approach for producing omega-3 fatty acids-enriched HON- and MAG-loaded nanoemulsions with physicochemical properties fully compliant with pharmacopeial standards for intravenous administration. The developed emulsions demonstrated excellent uniformity, strong electrostatic stabilization, and PFAT5 values comparable to those observed in clinically approved lipid emulsions such as Lipofundin and Lipidem. Importantly, the inclusion of HON and MAG did not compromise physicochemical quality; both compounds remained chemically stable during storage, and MAG enhanced physical stability under freeze–thaw stress, suggesting favorable interactions at the droplet interface.

The promising stability and biocompatibility, combined with the known hepatoprotective actions of HON and MAG, position these nanoemulsions as potential candidates for next-generation functional lipid systems for PN. Their application could be particularly valuable in preventing or mitigating IFALD, a major complication of long-term PN. The simplicity and industrial scalability of the production method further support their translational potential. Future in vivo and clinical studies will be crucial to confirm their safety, determine the optimal dosing, and assess their therapeutic impact in relevant patient populations.

## Figures and Tables

**Table 1:** Physicochemical properties of the developed nanoemulsions at t = 0 and t = 4 weeks of refrigerated storage.

Loaded Neolignan	t = 0				t = 4 Weeks			
	PDI	ZP	pH	OSM	PDI	ZP	pH	OSM
	Lipofundin-Based Nanoemulsions							
HON	0.050 ± 0.03	−43.1 ± 0.6	7.56 ± 0.1	340 ± 5	0.036 ± 0.03	−42.2 ± 1.6	7.63 ± 0.0	353 ± 5
MAG	0.057 ± 0.01	−43.6 ± 1.2	7.89 ± 0.1	356 ± 8	0.065 ± 0.01	−48.7 ± 1.2 *	7.80 ± 0.1	357 ± 10
HON+MAG	0.072 ± 0.00	−42.5 ± 1.7	7.96 ± 0.1	345 ± 6	0.067 ± 0.01	−42.2 ± 1.9	7.76 ± 0.1	349 ± 5
-	0.061 ± 0.01	−41.7 ± 0.5	7.77 ± 0.1	343 ± 6	0.068 ± 0.01	−46.6 ± 6.0	7.53 ± 0.0	345 ± 2
	Lipidem-Based Nanoemulsions							
HON	0.055 ± 0.01	−44.5 ± 1.0	7.63 ± 0.1	346 ± 5	0.054 ± 0.01	−44.9 ± 0.5	7.74 ± 0.1	364 ± 2
MAG	0.061 ± 0.02	−45.3 ± 0.1	7.94 ± 0.1	358 ± 6	0.058 ± 0.01	−49.5 ± 0.8 *	7.95 ± 0.1	354 ± 9
HON+MAG	0.053 ± 0.02	−44.8 ± 0.5	7.92 ± 0.1	350 ± 3	0.059 ± 0.01	−41.7 ± 3.8	7.77 ± 0.1	355 ± 4
-	0.069 ± 0.00	−43.7 ± 1.7	7.95 ± 0.0	349 ± 2	0.076 ± 0.00	−47.5 ± 1.4	7.69 ± 0.1	356 ± 14

Polydispersity index (PDI); zeta potential (ZP); osmolarity (OSM) * Statistically significant changes between t = 0 and t = 4 weeks (*p* < 0.05).

**Table 2:** The PFAT5 and EE% values of the nanoemulsions developed after 1, 2, and 3 freeze–thaw cycles.

Sample	1 Cycle		2 Cycle		3 Cycle	
	PFAT5 [%]	EE [%]	PFAT5 [%]	EE [%]	PFAT5 [%]	EE [%]
HON-loaded Lipofundin	0.025	84	0.022	81	**0.114**	82
MAG-loaded Lipofundin	0.049	100	0.007	100	0.012	100
HON+MAG-loaded Lipofundin	**0.436**	86 for HON 93 for MAG	**>1.0**	87 for HON 93 for MAG	**>1.0**	87 for HON 93 for MAG
Control-Lipofundin	0.005	–	0.009	–	0.025	–
HON-loaded Lipidem	0.019	90	**0.056**	91	**0.163**	87
MAG-loaded Lipidem	0.022	98	0.016	100	0.016	99
HON+MAG-loaded Lipidem	**0.587**	91 for HON 98 for MAG	**>1.0**	91 for HON 98 for MAG	**>1.0**	91 for HON 98 for MAG
Control-Lipidem	0.005	–	0.007	–	0.021	–

Encapsulation efficiency (EE%); percentage of lipid residing in globules larger than 5 µm (PFAT5). Values shown in bold indicate that the critical value was exceeded.

**Table 3:** Hemolysis percentage induced by HON- and MAG-loaded nanoemulsions.

Sample	Hemolysis [%]
HON-loaded Lipofundin	2.11
MAG-loaded Lipofundin	0.70
HON+MAG-loaded Lipofundin	1.41
Control-Lipofundin	1.41
HON-loaded Lipidem	0.70
MAG-loaded Lipidem	1.41
HON+MAG-loaded Lipidem	0.00
Control-Lipidem	2.11

**Table 4:** Results of PFAT5 values at t = 0 and t = 24 h in the compatibility studies of the selected PN admixtures with the developed nanoemulsion.

Sample	OSpec (t = 0 ⟶ t = 24 h)	LSpec (t = 0 ⟶ t = 24 h)	LPeri (t = 0 ⟶ t = 24 h)	KPeri (t = 0 ⟶ t = 24 h)
HON-loaded Lipofundin	0.0011 ⟶ 0.0003	0.0043 ⟶ 0.0085	0.0019 ⟶ 0.0016	0.0031 ⟶ 0.0066
MAG-loaded Lipofundin	0.0021 ⟶ 0.0008	0.0023 ⟶ 0.0026	0.0094 ⟶ 0.0033	0.0129 ⟶ 0.0377
HON+MAG-loaded Lipofundin	0.0254 ⟶ 0.0036	0.0029 ⟶ 0.0044	0.0029 ⟶ 0.0025	0.0172 ⟶ 0.0151
Control-Lipofundin	0.0009 ⟶ 0.0033	0.0009 ⟶ 0.0009	0.0014 ⟶ 0.0038	0.0059 ⟶ 0.0074
HON-loaded Lipidem	0.0021 ⟶ 0.0011	0.0019 ⟶ 0.0050	0.0034 ⟶ 0.0034	0.0066 ⟶ 0.0063
MAG-loaded Lipidem	0.0013 ⟶ 0.0005	0.0046 ⟶ 0.0050	0.0126 ⟶ 0.0066	0.0156 ⟶ 0.0110
HON+MAG-loaded Lipidem	0.0046 ⟶ 0.0018	0.0092 ⟶ 0.0082	0.0065 ⟶ 0.0054	0.0336 ⟶ 0.0064
Control-Lipidem	0.0031 ⟶ 0.0011	0.0007 ⟶ 0.0010	0.0012 ⟶ 0.0015	0.0169 ⟶ 0.0047

OSpec, Omegaflex Special EF; LSpec, Lipolfex Special; LPeri, Lipoflex Peri; KPeri, Kabiven Peripheral.

**Table 5:** Comparison of pH and osmolality values of PN admixtures before and after mixing with the developed nanoemulsions.

Type of PN	pH		Osmolality [mOsm/kg]	
	PN	PN with Lignan-Loaded Nanoemulsion	PN	PN with Lignan-Loaded Nanoemulsion
OSpec	5.63	5.58–5.69	1678	1663–1667
LSpec	5.35	5.31–5.36	1891	1860–1980
LPeri	5.24	5.25–5.27	936	918–928
KPeri	5.38	5.31–5.36	813	802–819

OSpec, Omegaflex Special EF; LSpec, Lipolfex Special; LPeri, Lipoflex Peri; KPeri, Kabiven Peripheral.

**Table 6:** Comparison of lipid phase composition in Lipofundin, Lipidem, and corresponding neolignan-loaded nanoemulsions.

Component	Lipofundin	Lipidem	Lipofundin-Based Nanoemulsions	Lipidem-Based Nanoemulsions
Soybean oil	50%	40%	25%	20%
MCT	50%	50%	50%	50%
Fish oil	-	10%	25%	30%

**Table 7:** Composition of pre-emulsion.

Components	HON-Loaded NE	MAG-Loaded NE	HON+MAG-Loaded NE	Neolignan-Free Control
	[g]			
MAG	-	2.8	1.4	-
HON	2.8	-	1.4	-
Fish oil	10	10	10	10
MCT	10	10	10	10
Egg yolk lecithin	1.2	1.2	1.2	1.2
Sodium oleate	0.05	0.05	0.05	0.05
Glycerol	2.25	2.25	2.25	2.25
Water	ad 100	ad 100	ad 100.0	ad 100.0

NE, nanoemulsion; MAG, magnolol; HON, honokiol; MCT, medium chain triglyceride.

## Data Availability

The raw data supporting the conclusions of this article will be made available by the authors on request.

## Supplementary Materials

The following supporting information can be downloaded at: https://www.mdpi.com/article/10.3390/molecules31111939/s1. Table S1: Results of physicochemical parameters of studied nanoemulsions after exposure to acid stress; Table S2: Results of physicochemical parameters of studied nanoemulsions after exposure to alkaline stress; Table S3: Results of physicochemical parameters of studied nanoemulsions after exposure to oxidative stress; Figure S1: Effect of HON-, MAG-, HON+MAG-Lipofundin nanoemulsions on THLE-2 cell viability after 24 h incubation, assessed by the MTT assay.
