# Supplementary material for: Development and Characterization of Intravenous Nanoemulsions Loaded with Magnolia officinalis Neolignans
Source: Molecules. 2026 Jun 3;31(11):1939. doi: 10.3390/molecules31111939 (PMC13257743; doi:10.3390/molecules31111939)
Supplement: Supplementary file 1 [file molecules-31-01939-s001.zip › molecules-4028839-supplementary.pdf]

Table S1. Results of physicochemical parameters of studied nanoemulsions after exposure to acid stress

Sample	MDD (t = 0)	PDI (t = 0)	ZP (t = 0)	pH (t = 0)	EE% (t = 0)
**HON-loaded Lipofundin**	2796.7	0.838	-6.3	0.59	90
**MAG-loaded Lipofundin**	738.9	0.533	-3.7	0.60	105
**HON+MAG-loaded Lipofundin**	433.4	0.490	-5.7	0.82	94 for HON<br>105% for MAG
**Control-Lipofundin**	872.5	0.662	2.4	0.61	-
**HON-loaded Lipidem**	2385.3	0.667	-8.0	0.56	91
**MAG-loaded Lipidem**	1798.3	0.691	-4.2	0.60	102
**HON+MAG-loaded Lipidem**	2851.1	0.971	-7.4	0.81	93 for HON<br>102 for MAG
**Control-Lipidem**	518.8	0.590	0.9	0.61	-

Table S2. Results of physicochemical parameters of studied nanoemulsions after exposure to alkaline stress

Sample	MDD (t = 0 → t = 168h)	PDI (t = 0 → t = 168h)	ZP (t = 0 → t = 168h)	pH (t = 0 → t = 168h)	PFAT (t = 0 → t = 168h)
**HON-loaded Lipofundin**	201.5 → **C**	0.069 → **C**	-67.5 → **C**	13.55 → 13.02	0.02 → **C**
**MAG-loaded Lipofundin**	192.1 → 177.3	0.073 → 0.059	-72.4 → -62.2	13.50 → 12.95	0.02 → 0.01
**HON+MAG-loaded Lipofundin**	198.1 → 188.2	0.064 → 0.063	-69.4 → -62.7	13.04 → 12.71	0.02 → 0.01
**Control-Lipofundin**	192.8 → **C**	0.057 → **C**	-70.2 → **C**	13.50 → 13.16	0.01 → **C**
**HON-loaded Lipidem**	189.6 → **C**	0059 → **C**	-67.4 → **C**	13.52 → 12.95	0.01 → **C**
**MAG-loaded Lipidem**	184.7 → 170.8	0.066 → 0.061	-70.8 → -60.2	13.53 → 12.84	0.02 → 0.01
**HON+MAG-loaded Lipidem**	182.9 → 174.1	0.061 → 0.059	-69.4 → -63.6	13.06 → 12.63	0.03 → 0.01
**Control-Lipidem**	187.4 → **C**	0.059 → **C**	-73.2 → **C**	13.54 → 13.15	0.01 → **C**

C - coagulation

Table S3. Results of physicochemical parameters of studied nanoemulsions after exposure to oxidative stress

Sample	MDD (t = 0 → t = 168h)	PDI (t = 0 → t = 168h)	ZP (t = 0 → t = 168h)	pH (t = 0 → t = 168h)	EE% (t = 0 → t = 168h)
**HON-loaded Lipofundin**	206.9 → 207.6	0.057 → 0.071	-43.2 → -33.0	3.88 → 3.90	85 → 85
**MAG-loaded Lipofundin**	206.9 → 204.1	0.056 → 0.058	-41.6 → -40.5	4.06 → 3.83	102 → 101
**HON+MAG-loaded Lipofundin**	210.9 → 211.8	0.055 → 0.064	-40.2 → -39.2	4.01 → 4.00	90 → 91 (HON)<br>103 → 104 (MAG)
**Control-Lipofundin**	200.5 → 196.3	0.054 → 0.066	-41.1 → -35.0	3.94 → 3.94	-
**HON-loaded Lipidem**	196.2 → 198.5	0.060 → 0.040	-43.0 → -38.0	4.14 → 4.22	92 → 91
**MAG-loaded Lipidem**	197.3 → 194.6	0.063 → 0.052	-43.3 → -42.9	4.34 → 4.16	101 → 100
**HON+MAG-loaded Lipidem**	194.7 → 196.0	0.049 → 0.047	-41.7 → -43.3	4.19 → 4.24	92 → 89 (HON)<br>107 → 103 (MAG)
**Control-Lipidem**	194.4 → 192.9	0.064 → 0.065	-41.4 → -41.3	4.27 → 4.24	-

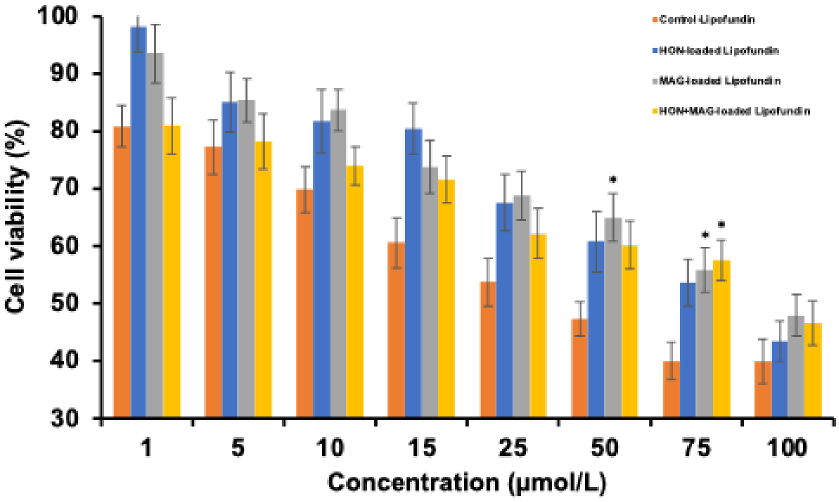

Figure S1. Effect of HON-, MAG-, HON+MAG-Lipofundin nanoemulsions on THLE-2 cell viability after 24-hour incubation, assessed by the MTT assay.

Data are presented as mean ± SEM (n = 3). * - statistical significance versus control was determined using Student's t-test ($p < 0.05$).
